# Assessing retention of pandemic capacities from COVID-19 in humanitarian settings: A cross-sectional retrospective study of health workers in Honduras, Syria, and South Sudan

**DOI:** 10.1371/journal.pgph.0006276

**Published:** 2026-04-20

**Authors:** Maryada E. Vallet, Husam Al Zuwayny, Christopher G. Kemp, Rose Lynn Cooper, Erin M. Sorrell, Gilbert Burnham

**Affiliations:** 1 TANGO International, Tucson, Arizona, United States of America; 2 Department of International Health, Johns Hopkins Bloomberg School of Public Health, Baltimore, Maryland, United States of America; 3 Independent Consultant, Tegucigalpa, Honduras; 4 Department of Environmental Health and Engineering, Center for Health Security, Johns Hopkins Bloomberg School of Public Health, Baltimore, Maryland, United States of America; Institute of Development Studies, UNITED KINGDOM OF GREAT BRITAIN AND NORTHERN IRELAND

## Abstract

The global health security landscape remains critically vulnerable to emerging pandemic threats. Humanitarian settings face particularly acute challenges in health workforce preparedness and response capabilities. Humanitarian settings have traditionally been left out of global health security and other health system strengthening investments. There is also a significant gap in the literature around building sustained health worker pandemic capacities in these challenging contexts. This novel study measured the retention or ‘shelf-life’ of perceived COVID-19 training benefits for health workers around knowledge, skills, and confidence to face future infectious disease threats. The Dynamic Sustainability Framework was used for conceptual framing. The study spanned three distinct humanitarian settings: Honduras, Syria, and South Sudan. Employing a cross-sectional, retrospective self-assessment design, 129 primary healthcare and community level health workers were surveyed in March-April 2024. Participants self-reported pandemic capacities on five-point Likert ratings across three time points – retrospective recall for pre- and post-training, and for present status, which was on average three years after training. Results demonstrated substantial increases post-training in self-reported knowledge (*p* < 0.001), skills (*p* < 0.001), and confidence (*p* < 0.001), and sustained or improved capacities at present (knowledge (*p* < 0.01), skills (*p* < 0.01), and confidence (*p* < 0.001)). Despite low access to ongoing training, resources, and support, 84.3% of health workers reported feeling prepared to face emergent disease risks (COVID-19 or other). The results call for further exploration of the individual, training-related, facility, and contextual factors affecting the capacity retention. Due to study design limitations, these results cannot be attributed to the trainings or generalized to all health workers in these countries. Still, this research contributes critical insights into the potential sustained benefits of frontline health workforce pandemic capacity building in humanitarian settings. Since capacities were retained despite limited ongoing training and support, targeted, sustained investments become crucial to preserve and enhance health security in the most fragile health systems.

## Introduction

Global Health Security (GHS) competencies require workforce capacity to rapidly respond to emerging health threats while safely performing regular duties [1]. The Sars-CoV-2 (or Coronavirus Disease 2019, COVID-19) pandemic precipitated an unprecedented mobilization of resources in humanitarian settings [2]. These investments rapidly developed capacity among frontline health workers to respond to infectious disease risks [3–5]. Yet, pandemic training supported by international assistance often lacks sustainable structures for ongoing local capacity building [6]. There is little known about the extent that pandemic-era capacities were enhanced and retained in humanitarian settings. It is widely agreed the world will face additional epidemics or pandemics in the years ahead, from diseases like Ebola Virus or from a novel human-to-human transmissible strain of avian influenza [7]. Some argue the COVID-19 pandemic has left the world less prepared [8]. This study measured the retention of health worker knowledge, skills, and confidence years after COVID-19 training. The study aims to inform GHS stakeholders and humanitarian partners on the continued investments needed to build from COVID-19 capacities and equip frontline humanitarian health workers for future health emergencies.

Nations experiencing persistent humanitarian crises have exceptionally dysfunctional healthcare infrastructure, systems, and vulnerable populations, limiting their capacity to address emergent disease threats [9–11]. These are contexts where emergencies have overwhelmed domestic response capabilities, either for the country as a whole or sub-nationally, necessitating international response [12]. Humanitarian assistance for the health sector is short-term and fragmented in nature, left out of long-term investment strategies for GHS [6], as well as for the interconnected prongs of health system strengthening and universal health care [13].

Published assessments of humanitarian health worker knowledge, attitudes, and practices during COVID-19 have been reported for Somalia [14], Uganda’s refugee-hosting regions [15], Rohingya refugee camps in Bangladesh [16], and conflict-affected areas of Ethiopia [17]. Broader studies including both humanitarian and low-resource settings spanned Latin America [18] and numerous African countries [5].

This study addresses literature gaps by assessing the level of retention of COVID-19 training outcomes (knowledge, skills, confidence) among healthcare workers in three countries: Honduras, Syria, and South Sudan. It utilizes an implementation science framework and outcomes to further measure adoption, penetration, equity, and sustainability of training benefits. The study is a cross-sectional, retrospective self-assessment of perceived capacity gains pre- to post-training and their sustainment over time. It moves beyond the immediate post-training assessments common in current literature [19] to analyze the extent that self-perceived outbreak or pandemic capacities were retained and institutionalized. For most health workers surveyed, nearly three years had passed since the trainings. This study was embedded within a larger COVID-19 Evaluation series (2020–2022) for the United States Agency for International Development/ Bureau for Humanitarian Assistance (USAID/BHA).

### Country context background

The study included Honduras, Syria, and South Sudan in order to represent one country from each USAID/BHA geographic region. The case study country selection was based on: high funding levels relative to that region; accessibility for survey teams; relevant sectoral funding related to health services; complex emergency designations (i.e., conflict and other concurrent crises); and implementing partner ability to participate. While distinct in their humanitarian challenges and contexts, they collectively provide insights into health worker capacity building during the pandemic.

#### Honduras.

Honduras’ centralized public health system has suffered from decades of low investment, resulting in limited laboratory services, weak infrastructure, and critical bed shortages [20]. During the pandemic, the system faced significant personal protective equipment (PPE) shortages and high rates of health worker infections [20]. In November 2020, Hurricanes Eta and Iota hit the northern highlands, affecting 4.7 million people, exacerbating vector-borne diseases like dengue, violent crime and insecurity, and leaving 500,000 without health services [21–23]. By March 2023, Honduras reported 472,250 COVID-19 cases and 11,111 deaths, with 66% of the population having received at least one vaccine dose [24]. At the time of this study, the global humanitarian appeal for Honduras included 1.3 million people [25]. The country ranked 163 of 195 in the GHS Index [26].

#### Syria.

From over a decade of conflict, an estimated half of Syria’s healthcare facilities were destroyed or damaged and 70% of healthcare personnel fled the country [27–29]. The pandemic further strained this system, which operated under fragmented control. At the time of data collection, the Syrian Arab Republic Government, shortened in this study to Government of Syria (GOS), controlled approximately 70% of the territory, and parts of Northern Syria (NS) were under various opposition control [27,30]. The Assad regime targeted health facilities and health workers in the opposition-controlled areas over the 13-year civil war, resulting in weaker infrastructure and systems overall [31,32]. In February 2023, an earthquake in northern and western Syria resulted in over 6,000 deaths [33] and complicated efforts to manage COVID-19 and other epidemic-prone diseases, such as cholera, meningitis, measles, and Hepatitis A [34]. The country reported 57,467 COVID-19 cases and 3,164 deaths by March 2023 [24], with only 13% of the population completing primary vaccination [35]. At the time of data collection, 16.7 million Syrians were in need of humanitarian assistance [33]. Syria ranked 192 of 195 in the GHS Index [26]. As of December 2024, a post-Assad unified Syria has the opportunity to rebuild the national health systems [36].

#### South Sudan.

South Sudan’s limited health system has been affected by insecurity and displacement crises since the country’s independence in 2011 [37,38]. Prior to the pandemic, an estimated 80% of medical services were provided by international organizations, and 67 of 80 counties lacked adequate health facilities [38–41]. Health workforce development mechanisms were also extremely limited [39,42]. Three consecutive years of flooding (2020–2022) deepened the humanitarian crisis, complicating COVID-19 management amidst severe food insecurity [37,43]. Concurrent outbreaks included widespread enteric fever (Typhoid), Hepatitis E virus, cholera, measles, and endemic malaria—a top killer of children under five [44]. The country reported 18,368 COVID-19 cases and 138 deaths by March 2023, with 28% of the population receiving at least one vaccine dose [24]. At the time of the survey, an estimated nine million people were in need of humanitarian assistance [45]. The country ranked 185 of 195 in the GHS Index [26].

## Materials and methods

### Design and instrument

The study employed a cross-sectional retrospective design to examine the self-reported retention of COVID-19 training outcomes among primary healthcare (PHC) workers in Honduras, South Sudan, and Syria. The study population comprised health workers who received capacity building support from humanitarian partners between 2020 and 2022. The sample frame included USAID/BHA-funded partners who implemented COVID-19 infection prevention and control (IPC)-related training and activities in PHC facilities during those years. For this study, a tailored survey module was included in the tool for the overall USAID/BHA COVID-19 Evaluation from which these data are drawn. There were no existing validated tools that aligned with the study objective and acceptable survey length, the humanitarian context, and the new COVID-19 realities, which limits the comparability to other research.

The study assessed training outcomes through self-reported measures of knowledge, skills, and confidence to face infectious disease threats at three time points: retrospective recall for pre-training (Time Zero, T0), retrospective recall for post-training (T1), and present status at time of data collection (T2). The three time points were gathered through one cross-sectional survey. Data collection was completed in South Sudan in March 2024 and in April 2024 for Honduras and Syria. This is nearly three years (34 months, median) following the COVID-19 trainings attended by the participants.

Self-assessment of psychometric constructs like knowledge, beliefs or attitudes, skills, and confidence is common in medical education [46]. For the outcome variables, a five-point rating scale was used to reduce “decision noise” [47]. The tool included open-ended questions that were thematically coded as structured responses for the proxy outcomes of sustainability and equity. Thematic coding was conducted by two study team members separately, with differences discussed and agreed with the study team leader.

#### Conceptual framework.

Table 1 provides a conceptual framework with a summary of the implementation science concepts guiding the study [48]. It shows how the implementation strategy, implementation outcomes, service outcomes, and training outcomes are related to the overall pandemic preparedness goal. Adapted from the Proctor et al. definitions [48]: Adoption is the initial intention/uptake of the practice, measured here through post-training assessment; Penetration is integration in the service setting, measured as refresher training or supervision; Sustainability refers to the extent of facility institutionalization, measured as health worker perceptions of feeling supported by the facility to face future infectious disease risks. This study was informed by other frameworks as well. From implementation science literature, the Dynamic Sustainability Framework emphasizes the sustainability of benefits over time [49]. Additionally, key study measures align with the Kirkpatrick four-stage model to measure reaction, learning, behavior, and organization results: satisfaction, knowledge, skills, confidence, and application [50]. The Kirkpatrick Model is commonly used for evaluating learning and training activities and has been applied to health worker in-service trainings in various contexts [51–53].

**Table 1:** Implementation Science Conceptual Framework.

Evidence-based Practice	Implementation Strategies	Implementation Outcomes	Service Outcomes	Pandemic Training Outcomes	Pandemic Preparedness Goal
Building health worker capacity in IPC, etc.	COVID-19 frontline health worker **in-service training** (varied by delivery modality, duration, and topics covered)	**Adoption**: initial uptake through post-training assessment **Penetration**: refresher training or supervision **Sustainability**: facility-level support	**Equity**: Gaps in perceived preparedness differing by group **Satisfaction**: Health worker satisfaction with training	Increased health worker pandemic capacity: **knowledge**, **skills**, and **confidence**	**Readiness maintained** among PHC workforce to control future infectious disease threats

#### Expert and ethical review.

The survey instrument underwent review by evaluation, implementation research, and humanitarian health experts. It was translated into Spanish (Honduras), Arabic (Syria), and maintained in English (South Sudan), employing a dual forward translation process with in-country validation. Tools were pilot tested with local health professionals in each context before programming with Open Data Kit (ODK)/Kobo Collect for tablet-based data collection.

The Johns Hopkins Bloomberg School of Public Health Institutional Review Board (IRB) Office reviewed all study procedures and provided IRB determination that this is not human subjects research. Verbal consent was gained for all survey participants. The consent statement was read to participants and they were asked to summarize research activities to confirm understanding of the procedures, which was witnessed and documented by the survey enumerator. Study activity approvals were received by USAID staff representing each country, who confirmed the study did not require national IRB and provided country-level oversight. Study approvals and coordination were also sought with local authorities and participating ministries through the local research partners research partners ANED Consultores (Honduras), Trust Consultancy and Development (Syria), and Specialized Logistics Solutions (South Sudan).

**Inclusivity in global research**: Additional information regarding the ethical, cultural, and scientific considerations specific to inclusivity in global research is included in the Supporting Information (S1 Checklist).

#### Sample selection.

The sample size follows the Cochran calculation for a single proportion, appropriate for ordinal outcomes in descriptive cross-sectional surveys [54,55]: using knowledge, skills, and confidence outcomes on five-point scale. Assuming maximum variability (*p* = 0.50), 95% confidence level (Z = 1.96), and a 10% margin of error (e = 0.10), the minimum required sample size was 96 participants.

Each country aimed for 30–35 total surveys across the selected sites, the generally agreed minimum for subgroup disaggregation [56]. Due to the different governance and operational contexts within regions of Syria controlled by the regime (GOS) versus other authorities (NS), these two regions were sampled separately. The non-probability sampling strategy included purposively selecting partner health facility sites and then identifying health worker participants across those sites to total approximately n = 30. This approach allowed for capturing a snapshot of the workforce dynamics and conditions in each country. The case study country selection is described above, see Country context background.

The health facility sites were selected purposively with the implementing partners in each country/location based on logistical and security feasibility. Then, the participant selection criteria required that the health worker worked in community/primary health services and received COVID-19 training from 2020-2022. For each site, it was determined how many health workers were trained and how many were still available at the facility. If a census of those workers was not possible because more were available than needed for the sample, they were chosen randomly from those available at the time of data collection. The study minimized the risk of selection bias by comparing key characteristics of the trained health workers who were not available to the survey participants to ensure no difference based on individual traits such as role, gender, or ethnicity. This potential bias in the sampling is further addressed in the limitations.

Due to local conditions and religious holidays, the survey recruitment varied by country case study, start and end dates included: 12/03/2024 – 18/04/2024 for Honduras; 04/04/2024 – 30/04/2024 for Syria; 16/03/2024 – 28/03/2024 for South Sudan. To ensure safety, the separate Syria teams ensured anonymity of the researchers, partners, and participants across the regions. All survey data remained confidential and was uploaded to secure servers.

### Analysis procedures

Data collected using ODK survey software were exported into a secure database across the countries. Then data were downloaded into Excel for cleaning and preliminary descriptive analyses. StataSE 18 64-bit software was used for descriptive and statistical analyses [57]. As the sample size was not calculated for country-level differences, nonparametric testing in StataSE was used to assess the significance of the differences. Wilcoxon non-parametric tests are common for testing Likert scale outcomes such as with retrospective pre-test methods [58–60]. The Wilcoxon Sign-Rank Test assumptions were not fully met. Thus, the Wilcoxon Sign Test was selected because it can handle ties effectively, small sample sizes, and does not require a specific distribution.

The Wilcoxon Sign Test was performed to assess whether the median of the differences between within subject paired observations of self-rated knowledge, skills, and confidence to face another pandemic is significantly different from zero (from T1 to T0; T2 to T1). After data collection, the adequacy of the original sample size for the Sign Test and for country disaggregation was assessed. A power analysis was conducted based on the proportion of change in matched pairs. There is sufficient power to detect T1-T0 changes overall and by country, though some T2-T1 comparisons disaggregated by country were underpowered. For this reason, only overall results for the Sign Test are presented below.

## Results

### Sample overview

Table 2 shows the final survey sample of 129 health workers (HW) by country/location, number and type of partner (United Nations (UN), Government, or Non-Governmental Organizations (NGO)), and number of health facilities.

**Table 2:** Final Sample Overview.

Country/Location (sub-regions or states)	Type of partner	Number of projects	Number of health facilities	n=
**Honduras** (Atlántida, Cortés, Yoro)	UN/Government of Honduras	1	15	32
**Syria: GOS** (Hama and Damascus Governorates)	UN	3	8	36
**Syria: NS** (Hasaka, Idleb, northern Aleppo)	UN	3	6	39
**South Sudan** (Upper Nile and Warrap States)	NGO	2	11	22
**Total sample:**		9 Projects	40	129

The Honduras survey included health workers across eight departments of three regions, the majority in Cortes, a region heavily impacted by the hurricanes. The UN project in Honduras supported Government of Honduras PHC facilities, with this survey comprising 32 workers across 15 health centers. The survey in Syria included 39 workers of six facilities from NS territories and 36 workers from eight facilities from GOS, covering projects by three UN agencies implemented through local partners. South Sudan interviews covered two remote project areas of international NGOs. Due to high levels of displacement from flooding and insecurity, the aim of 30 interviews was not reached; the 22 interviews represented all available health workers meeting the criteria.

Overall, the health workers were majority female (60.5%) and serving in clinical roles (59.7%) during the COVID-19 response (Table 3). However, the NS and South Sudan samples were majority male, and for NS, most served in non-clinical roles. Approximately half (48.4%) of health workers had not finished university education, and this proportion was highest in South Sudan (85.7%). The GOS sample possessed the most education. The average years of experience in the healthcare field for participants was 9.1 years.

**Table 3:** Surveyed Health Worker Profile Across Case Countries.

Country/Location (n)	Female (%)	Clinical role (%)	No university degree (%)	Years of experience (mean [range])
**Honduras (32)**	93.8	78.1	43.8	12.3 (1-40)
**Syria: GOS (36)**	75.0	75.0	25.0	10.2 (3-36)
**Syria: NS (39)**	38.5	35.9	53.8	7.0 (0-29)
**South Sudan (22)**	27.3	50.0	85.7	6.2 (1-15)
**Total (129)**	60.5	59.7	48.4	9.1 (0-40)

### Training characteristics

Table 4 shows overall training characteristics related to modality and duration, as recalled by the surveyed health workers. The most common training modality was in-person (53.4%). Health workers reported a wide spectrum of training duration, with the most common response for 1–2 days of training (33.0%), followed by 3–5 days (26.3%). The health workers reported which topics were covered in the COVID-19 trainings.

**Table 4:** Percent of Health Workers Reporting Training Modality and Duration.

Recalled Training Characteristic	Overall
Percent of health workers by reported training modality:	In person: 53.4 Remote: 25.4 Hybrid: 21.2
Percent of health workers by reported training duration:	Less than one day: 14.4 1-2 days (up to 16 hours): 33.0 3-5 days (up to 40 hours): 26.3 6-10 days (up to 80 hours): 14.4 >10 days: 11.9
*n*	*118*

As shown in Table 5, proper use of PPE, COVID-19 transmission routes, IPC procedures in the health facility and in the community, and case reporting were the most commonly recalled training topics. Health workers recalled nine topics on average.

**Table 5:** Percent of Health Workers Reporting COVID-19 Training Topics by Location.

Recalled COVID-19 Training Topic	Syria: GOS	Syria: NS	Honduras	South Sudan	Total
Proper use of PPE	63.9	100.0	100.0	85.0	86.4
COVID-19 transmission routes	41.7	84.6	100.0	90.0	75.4
IPC procedures in the health facility (handwashing, triage, isolation)	50.0	94.9	91.3	65.0	75.4
IPC procedures in the community or schools	44.4	87.2	91.3	50.0	68.6
Case reporting to the health system (Ministry of Health)	19.4	66.7	91.3	70.0	57.6
Risk Communication and Community Engagement (RCCE) and addressing rumors or misinformation	38.9	56.4	82.6	55.0	55.9
COVID-19 vaccination dissemination procedures	19.4	59.0	95.7	65.0	55.1
Procedures for continuing basic health/nutrition services during pandemic	33.3	56.4	87.0	50.0	54.2
Disinfection and waste management in health facility	25.0	64.1	87.0	45.0	53.4
Case management (home-based care, treatment referral)	16.7	61.5	91.3	60.0	53.4
Case detection/screening in the health facility	19.4	38.5	82.6	90.0	50.0
Community surveillance	27.8	43.6	87.0	50.0	48.3
Mental health and support for frontline workers	52.8	33.3	52.2	35.0	43.2
Identifying vulnerable individuals in health facility or community (gender-based violence, food insecurity)	13.9	35.9	87.0	40.0	39.8
Supply chain (procedures for inadequate PPE supply)	0.0	38.5	87.0	50.0	38.1
Case testing/laboratory procedures	8.3	20.5	78.3	35.0	30.5
Other	2.8	2.6	4.3	5.0	3.4
**Mean number of topics covered per training**	**4.8**	**9.4**	**14.0**	**9.8**	**8.9**
*n*	36	39	23	20	118

#### Implementation and service delivery.

Proxy measures were developed for the study’s conceptual framework constructs around implementation and service outcomes, including adoption, penetration, sustainability, equity, and satisfaction [48]. Table 6 shows descriptive results of these measures overall and by country. Most (86.4%) health workers reported a post-training assessment (adoption). Just over half (57.2%) reported ongoing refresher training and/or supervision as follow-up to the COVID-19 training (penetration). Still, four in five (84.3%) reported feeling supported to face future outbreaks, indicating some institutionalization at the facility level (sustainability). Health workers across Syria (GOS and NS) may be more likely to report gaps in their knowledge or skills to handle future outbreaks or pandemics (equity). Finally, most health workers (83.8%) across locations were satisfied or very satisfied with the COVID-19 trainings (satisfaction).

**Table 6:** Adoption, Penetration, Sustainability, Equity Measures.

	Overall (%)	Honduras (%)	*Syria: GOS (%)*	*Syria: NS (%)*	South Sudan (%)
**Adoption: Percent of HWs reporting initial uptake through recall of post-training assessment**					
Yes	86.4	65.2	86.1	89.7	95.0
No	10.2	26.1	8.3	5.1	5.0
Do not recall	3.4	8.7	5.6	5.1	0.0
*n*	*118*	23	36	39	20
**Penetration: Percent of HWs reporting a refresher or mentoring follow-up to the training (health worker level institutionalization)**					
No, none	37.3	17.4	47.2	35.9	45.0
Refresher training	18.6	4.4	19.4	20.5	30.0
Direct supervision, mentoring, or hands-on feedback	26.7	43.5	22.2	23.1	20.0
BOTH refresher and direct supervision	11.9	26.1	11.1	7.7	5.0
Do not recall	5.9	8.7	0.0	12.8	0.0
*n*	118	23	36	18	20
**Sustainability: Percent of HWs reporting they feel supported by their health facility/organization to be prepared for a future outbreak or pandemic (facility level institutionalization)** ^**a**^					
Not supported	5.5	15.6	0.0	2.6	5.0
Somewhat supported	10.2	15.6	16.7	5.1	0.0
Yes, supported	84.3	68.8	83.3	92.3	95.0
*n*	127	*32*	*36*	*39*	*20*
**Equity: Percent of HWs reporting specific gaps in their knowledge or skills to handle future outbreaks or pandemics** ^**a**^					
No/Don’t think so	31.8	6.3	47.2	46.2	18.2
Yes, Gaps reported	68.2	93.8	52.8	53.9	81.8
*n*	127	*32*	*36*	*39*	*22*
**Satisfaction: Percent of HWs reporting overall satisfaction with COVID-19 training received**					
Not satisfied	1.7	4.4	2.8	0.0	0.0
A little satisfied	13.6	8.7	36.1	2.6	0.0
Satisfied	46.2	43.5	52.8	43.6	42.1
Very satisfied	37.6	43.5	8.3	51.3	57.9
*n*	117	23	36	39	19

^a^Open-ended question thematically coded to categories presented.

### Results by capacity

The pandemic capacities (knowledge, skills, confidence) are presented next. For each, descriptive results are presented, showing the percent of health workers by self-rated level and country/location. That is followed by a table providing evidence of overall self-assessed change across the time points.

**Fig 1:**
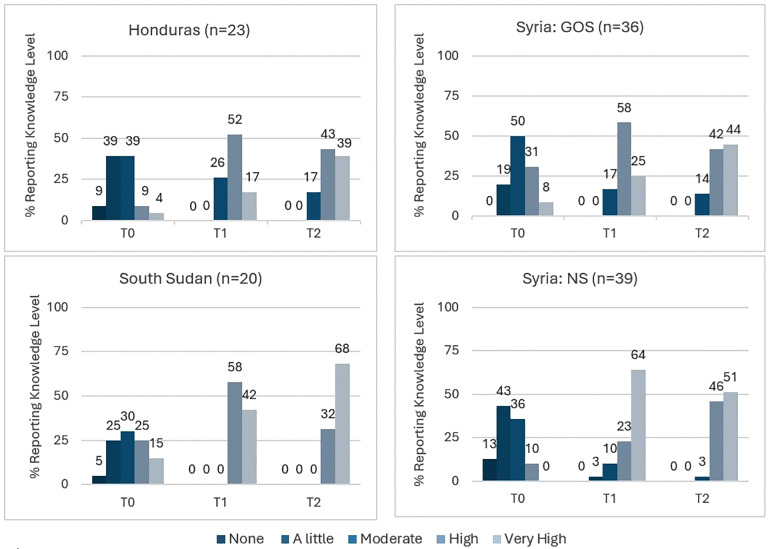
Percent of Health Workers Reporting Knowledge Level, by Time and Location.

**Knowledge**: It should be noted that the health workers were asked about knowledge on IPC-related training topics, not specifically about COVID-19 disease information. Fig 1 shows the percentage of health workers by self-rated knowledge level over the three time points by location. While workers reported no knowledge of IPC-related topics pre-training (T0) in Honduras, South Sudan, and NS, this category shifted toward higher ratings in other time points. At the time of the survey, no health workers report “none” or “little” knowledge. All GOS health workers reported at least “a little” IPC knowledge prior to COVID-19 trainings; this sample also had high levels of advanced education (Table 3). Over two-thirds of the health workers in South Sudan (68%) rate their current (T2) knowledge level of IPC-related topics as very high. As shown in the country profiles of Table 3, the South Sudan sample was the least educated, with more male health workers, and less experience compared to the other locations. There is an upward trend for the “very high” knowledge rating category across time points for all locations, except for Northern Syria. In NS, 64% of surveyed health workers reported having “very high” knowledge after the training but that drops to 51% years later at the time of the survey. The NS sample also differs from the other locations with the majority of health workers in non-clinical roles (Table 3).

**Fig 2:**
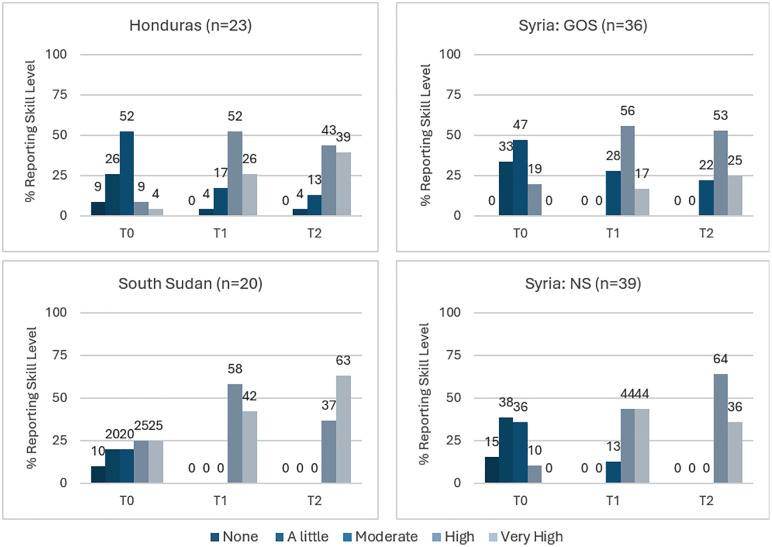
Percent of Health Workers Reporting Skill Level, by Time and Location.

**Skills**: Fig 2 shows the overall shift of health workers’ perceived improvement in IPC skills related to new infectious disease risks from 2020 to 2024 (pre-training to time of survey). While most health workers did not rate their current level of skills as very high, there is a clear trend away from low to moderate skills over the time points. The exception, again, is the health workers from South Sudan, with 63% reporting “very high” skills at the time of the survey. Similar to the knowledge results, no health workers report their skills as “none” at the time of survey (T2). All GOS health workers reported at least “a little” relevant skill pre-pandemic training, which could reflect their higher education and clinical training (Table 3).

**Fig 3:**
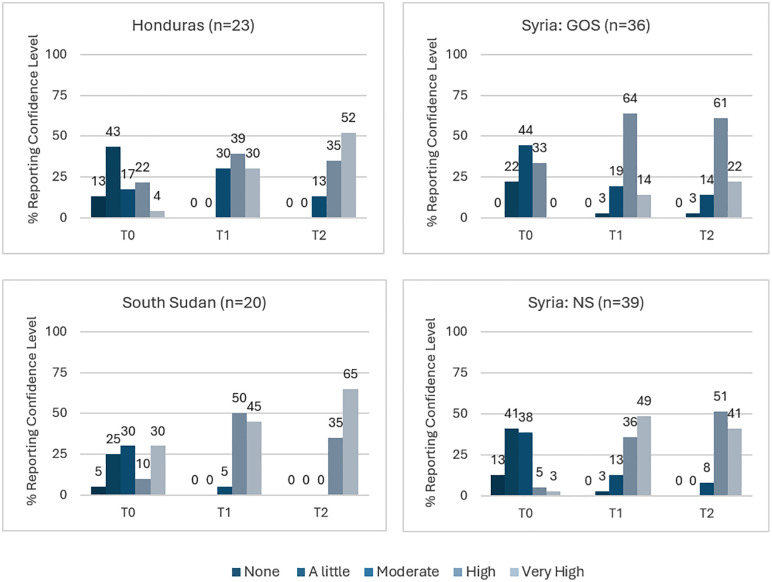
Percent of Health Workers Reporting Confidence Level, by Time and Location.

**Confidence**: Health workers then rated their confidence to apply their knowledge and skills to future infectious disease risks (Fig 3). The trends for knowledge and skills above remain in terms of the shift from no or very low confidence pre-training (T0) to higher levels after years of pandemic training and experience.

### Overall sustained capacity

Table 7 shows the median health worker rating, interquartile range (IQR), and significance level for knowledge, skills, and confidence over the recalled time points. There is a highly significant (*p* < 0.001) change reported from pre-training to post-training across the capacities. This perceived improvement in pandemic capacities continues overall from post-training to the time of data collection.

**Table 7:** Median [IQR] Health Worker Self-rated Capacities Over Time.

Change Over Time	Knowledge (n = 118)	Skills (n = 118)	Confidence (n = 118)
Pre-training recall (T0): Percent rating “Very High”	3.4%	5.1%	6.8%
Post-training recall (T1): Percent rating “Very High”	39.3%	31.6%	33.9%
Present/Time of survey (T2): Percent rating “Very High”	49.6%	37.6%	41.5%
**Pre to Post-Training *(T0 - T1)***	4.0 [4.0-5.0]***	4.0 [4.0-5.0]***	4.0 [4.0-5.0]***
**Post-Training to the Present (*T1 - T2)***	4.5 [4.0-5.0]**	4.0 [4.0-5.0]**	4.0 [4.0-5.0]***

*p < 0.05, *p* < 0.01 **, *p* < 0.001 ***.

## Discussion

### Overview

The results show significant improvements in humanitarian health workers’ perceived pandemic capacities following COVID-19 trainings in Honduras, Syria, and South Sudan. The capacities - knowledge, skills, and confidence to face emerging infectious disease risks – increased post-training and continued to improve for years until the survey. Overall satisfaction with COVID-19 training was high. Training format, duration, and topics varied widely [61]. The findings suggest that COVID-19 training for health workers primarily relied on short, face-to-face training approaches rather than extended or fully remote capacity-building during the response period. The former trainees recalled nine training topics on average. The most common recalled training themes were: proper use of PPE, transmission routes for respiratory disease, IPC procedures in the facility and community (e.g., screening, isolation, handwashing, etc.), and Risk Communication and Community Engagement (RCCE) approaches. These training topics are applicable to many other disease threats faced by humanitarian health workers, from other SARS viruses to direct contact, water or vector-borne diseases [61].

While implementation outcomes showed strong initial uptake (i.e., post-training assessment), penetration through follow-up training was notably lower. This is likely related to decreased pandemic-related funding by the end of 2022 and the lack of long-term health workforce development financing [62]. Despite the limited refresher training, most health workers reported feeling supported by their health facility or organization to face future outbreaks or pandemics. This assessment did not explore the psychosocial or other reasons the health workers felt supported despite actual resource and training shortages, which would be an important area for additional research.

The post-training results overall resonate with literature on post-training assessment of health workers’ perceived knowledge, skills, and confidence to handle COVID-19 cases [5]. An unexpected finding from this study is that these capacities were perceived as sustained and increased over years since the training. This, with only moderate levels of refresher supports along with equipment and resource shortages. Existing literature on COVID-19 and prior outbreaks or epidemics in humanitarian settings highlights consistent resource constraints that limit ongoing training, supervision, and ability to enact IPC knowledge (i.e., lack of access to PPE) [5,15,63]. Some differences were also observed by location, which may be related to health worker education, role, gender, experience, and other characteristics. These individual, contextual, and facility-level factors that contribute to sustainment of capacities should be further studied. While more research is needed, to the authors’ knowledge, no prior studies have measured the retention—or ‘shelf-life’—of pandemic preparedness capacities among humanitarian health workers following COVID-19 or health emergency–related training interventions.

### Discussion by capacity

The limited literature that examined COVID-19 and IPC-related knowledge increases among health workers in humanitarian contexts showed similar low-to-moderate knowledge levels in the first year of the pandemic as this study’s pre-training levels. In Somalia and in Uganda’s refugee-hosting region, health workers surveyed mid-2020 exhibited low general knowledge of COVID-19 [14,15]. A worldwide health worker e-survey in March 2020 showed similar trends, finding low knowledge overall, and social media was a major source of early COVID-19 information [64]. It also found that different age and roles were associated with inadequate knowledge and a poor understanding of COVID-19 [64]. This may explain higher pre-pandemic knowledge, skills, and confidence for GOS health workers, as they had the highest levels of education and more years of experience. Whereas the higher concentration of “very high” self-ratings observed in the South Sudan sample across the capacities (T2) may reflect differences in training. Per the Dunning-Kruger effect, individuals with lower levels of education or experience may overestimate their performance [65].

Overall, health workers were able to recall training specific to COVID-19 transmission and many critical IPC-related topics, with proper use of PPE standing out among these frontline workers. This aligns with global evidence showing that health workers who received COVID-19 training reported greater IPC and disease-specific awareness, underscoring the value of just-in-time training for improving knowledge [4]. Access to PPE is further discussed below as it relates to health worker skills.

The limited literature from humanitarian contexts on skill development during the pandemic generally links improved IPC practice with access to adequate training and PPE [17]. WHO facility-level surveys conducted January 2021-March 2022, including 15 countries with humanitarian crises, showed just 53% of PHC facilities with adequate PPE [66]. For humanitarian settings, where frontline primary healthcare resources are limited and rely on non-clinical and community workers, it is particularly important to include training on handling situations of limited PPE supply and on proper usage of PPE [61]. This reveals the challenges of supporting staff to mitigate risk with proper IPC practices during the COVID-19 response and the importance of ongoing pandemic capacity building.

This study shows significant increases in health workers’ perceived ability to apply the knowledge and skills to future outbreaks or pandemics, resonating with literature showing that confidence or reported readiness during COVID-19 rose with training [5,15]. Other studies among frontline health workers in Ethiopia, Uganda, and globally have linked COVID-19 trainings around case management and IPC with less anxiety and more perceived ability to properly handle cases [4,67,68]. Health workers who were aware of their facility’s safety and risk management plans and had access to PPE also demonstrated higher confidence to face the pandemic in a study across Latin America, including some humanitarian settings [18].

### Policy implications

This study shows that GHS investments in countries with humanitarian contexts and fragmented systems can indeed have sustained impacts. It also shows the importance of building capabilities at the frontlines, including among community health workers [13]. Yet, these contexts have historically been left out of GHS strategies and investments.

By way of demonstrating this gap, at the end of 2022, the COVID-19 global UN humanitarian appeal, based on direct and indirect pandemic effects, identified South Sudan as the country with the largest percentage of population in need of assistance (75%), followed by Syria (69%); Honduras was in the top 14 countries at 31% [69]. These countries were not part of the U.S. GHS intensive support countries at that time [70]. It was promising when Honduras and South Sudan were added with the U.S. Global Health Security Strategy expansion of April 2024 [71]. However, the Trump Administration’s cessation of USAID assistance and of the 2024 Strategy in early 2025 has severely halted that progress [72]. The new U.S. Global Health Strategy of 2025 includes a focus on pandemic prevention and preparedness primarily through bilateral agreements [73]. It is yet to be seen if this strategy will reach isolated and fragmented humanitarian health systems, because GHS policy implementation focuses on national systems.

The International Health Regulation (IHR) 2005 is the legal instrument adopted by World Health Organization (WHO) Member States to strengthen country-level GHS capabilities to prevent, detect, and respond to health emergencies [70]. This policy, and its monitoring and evaluation tools for tracking progress, have lacked applicability to humanitarian settings due to its reliance on national systems and policies for implementation. In humanitarian settings, the application of IHR core capacities is challenged by dysfunctional or nonexistent governance structures [74,75], insufficient data availability for measuring outcomes [76,77], and humanitarian actors not aligned with the IHR where limited systems do exist [78]. The IHR does not currently support a system for countries with widespread complex emergencies and inoperable sub-national health systems. IHR amendments in mid-2024 provided agreement around key themes that may better support the most fragile contexts, including the focus on equity and a new coordinating financial mechanism [79]. Building on this momentum, the historic Pandemic Agreement of 2025 could provide a way-forward to address these gaps as it contains mention of humanitarian settings and an emphasis on preparedness through the primary health-care approach [80].

Finally, the findings of this study may help sharpen the focus of the IHR, GHS, and resilient health systems agendas for frontline health workers in fragile settings [81]. Although COVID-19 brought an unprecedented surge of resources that temporarily strengthened health workers’ knowledge, skills, and confidence to manage infectious disease threats, important gaps remain. Evidence from humanitarian contexts—from Yemen to Haiti—shows that effective pandemic preparedness depends on foundational investments in health infrastructure, workforce development, coordination, and sustained funding [82,83]. Strengthening community- and facility-level case detection and IPC capacity, beyond specialized epidemiologic teams, is also essential to safeguard frontline environments [6,84–86]. Capacities built at the PHC level underpin health system resilience and advance universal healthcare and system-strengthening agendas [13,87–90]. There is a need for integration of these goals across the global health architecture [91]. This research adds further evidence supporting these critical policy and investment priorities.

### Limitations

There are various limitations to note in the interpretation of these findings, both in terms of study and survey design. Importantly, the non-probability sampling limits generalizing the results to the population, and the non-experimental design precludes attribution of results to the training intervention. There is the potential for selection bias due to the purposive site selection, as well as loss to follow-up and staff turnover in humanitarian contexts, which was over 50% of trained staff in NS and South Sudan. This may have influenced the sample by potentially over-representing accessible health facility sites and retained health workers with higher capacity. To mitigate this risk, the study tracked total trained health workers across sites. Comparative analyses of worker characteristics (e.g., education level, role, vulnerability to other crises) were conducted with local partners to verify representativeness of the remaining workforce. And of that remaining workforce at selected sites, the sample was either a census or random selection among available staff. Further, the lack of a standardized tool limits comparability to other literature. In terms of the analyses, the Wilcoxon Sign Test has less statistical power to detect differences than the Signed-Rank Test, for which assumptions were not met. While the Sign Test is valid in small samples, these findings should be interpreted cautiously.

Next with regard to cognitive biases, recall and positivity bias in self-assessment are challenges [92]. Other biases include stability bias for medical professionals’ self-assessment, and the Dunning–Krueger effect, in which lower-skilled professionals overestimate their performance [65]. To mitigate these, the interviews were conducted with privacy and anonymity assured. Interviewers continuously reminded the respondent about the time period and training project under scope using key pandemic timepoints and project descriptions to address recall. While all recall bias cannot be ruled out, some literature provides justification for this study’s retrospective recall measures using longer-term self-assessment. These can be more accurate based on the health professionals’ overall experience and understanding of their pre- and post-training changes over time, related to response shift bias [65,93,94].

## Conclusions

This study offers preliminary evidence that frontline health workers in Honduras, Syria, and South Sudan were able to sustain—even strengthen—their pandemic capacities in the years since COVID-19 pandemic training and response (2020–2024). These findings address a notable gap in the literature on the durability of health emergency competencies built from in-service training in humanitarian settings. Although the study’s design does not allow attribution to specific trainings or generalization to broader populations, it highlights critical avenues for further investigation. Future studies should examine the gaps that persist in health worker pandemic capacity, as well as the contextual, facility-level, training-specific, and individual factors that shape capacity sustainment.

While further research is needed to better understand the factors shaping capacity retention, these findings already point to a clear reality: frontline health workers have sustained pandemic capacities despite limited institutionalization of preparedness support within their facilities. To preserve and expand these capacity gains, targeted and sustained investments in humanitarian health systems and the health workforce are crucial. This includes continuous professional development, peer mentoring, refresher training, and supportive organizational systems at the primary healthcare level. For humanitarian organizations, this includes designing pathogen-agnostic training approaches aligned with International Health Regulation competencies, rather than single-pathogen responses. It also requires building internal mentorship and supply chain structures that can sustain skills despite high staff turnover and funding volatility. Such investments should be embedded within the financing mechanisms for the International Health Regulations, the Pandemic Agreement, and Universal Health Care.

These findings underscore the responsibility of global health security strategies to better account for frontline health workers operating in low-governance, sub-national humanitarian health systems. As the global health architecture evolves in future years, dedicated and context-tailored policies and financing for primary healthcare and humanitarian contexts will be crucial. This is not only to protect the COVID-19 capacities already built, but to match the demonstrated commitment of these health workers to health security and preparedness for future pandemics.

## Supporting information

S1 Data Health worker survey data. The underlying dataset anonymized.
(XLSX)

S1 Checklist Inclusivity in global research. Questionnaire.
(DOCX)
